# Improving tumor treatment: Cell membrane-coated nanoparticles for targeted therapies

**DOI:** 10.1016/j.mtbio.2025.101716

**Published:** 2025-04-23

**Authors:** Pablo Graván, Juan Antonio Marchal, Francisco Galisteo-González

**Affiliations:** a Department of Applied Physics, University of Granada, Avenida Fuente Nueva, s/n, Granada, 18071, Spain; b Excellence Research Unit Modelling Nature (MNat), University of Granada, Avenida Fuente Nueva, s/n, Granada, 18016, Spain; c Department of Human Anatomy and Embryology, Faculty of Medicine, University of Granada, Granada, 18016, Spain; d Instituto de Investigación Biosanitaria ibs.GRANADA, Granada, 18012, Spain; e Biopathology and Regenerative Medicine Institute (IBIMER), Centre for Biomedical Research (CIBM), University of Granada, Granada, 18016, Spain; f BioFab i3D- Biofabrication and 3D (bio)printing laboratory, University of Granada, Granada, 18100, Spain

**Keywords:** Cell membrane-coated nanoparticles, Cancer, Drug delivery, Phototherapy, Immunotherapy, Isolation procedures

## Abstract

Cells membrane-coated nanoparticles (CMs-NPs) represent a highly promising platform in cancer treatment. Due to the various types of cell sources employed and the broad designs of NPs, CM-NPs have emerged as versatile and multifunctional platforms with wide applicability in medicine. This literature review showcases the applications of CMs-NPs in cancer therapy, highlighting significant advancements in tumor-targeted delivery, phototherapy, and immunotherapy. Different cell types employed as CMs sources are reviewed, including cancer cells, red blood cells, platelets, white blood cells, stem cells, fibroblasts, and bacterium. Hybrid CMs-coatings and the technology to produce them are also included. Additionally, the state of the art in methodologies is critically examined, noting that while effective methods for coating and isolation of CMs exist, further optimization is still required. The latest reports and research findings in this regard are also presented, emphasizing the continuous need for innovation to overcome substantial challenges related to this promising nanotechnology. The aim of this review is to provide an in-depth overview of the evolving landscape in the development of effective and targeted cancer treatments, underscoring the transformative potential of CMs-NPs in revolutionizing cancer care and improving patient outcomes.

## Graphical abstract

**Figure:**
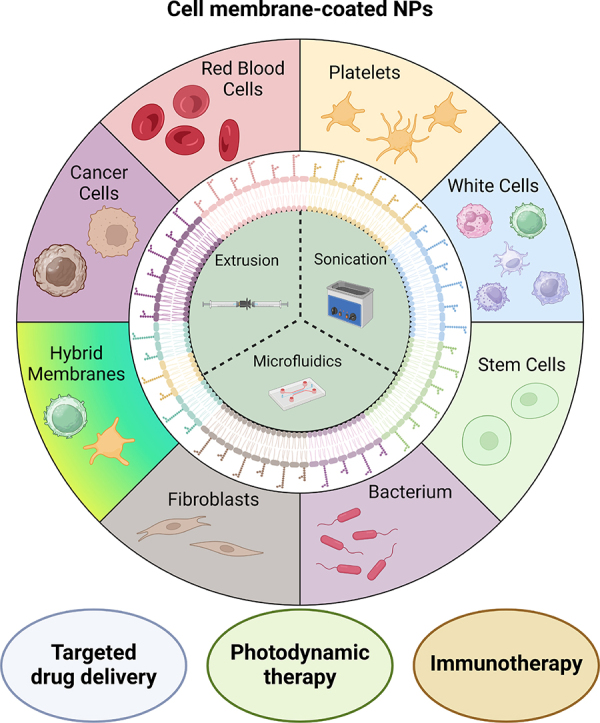

## 1 Introduction

Cancer remains a major challenge in the 21st century, significantly impacting public health and the global economy [1]. In 2022 alone, nearly 20 million new cases were recorded worldwide, with statistics suggesting that approximately one in five individuals will develop cancer during their lifetime, and a significant number will succumb to it [1]. Cancer encompasses a heterogeneous group of disorders characterized by abnormal and uncontrolled cell growth. It can manifest in any part of the body, with surgical resection, chemotherapy, and radiotherapy serving as the three main conventional treatment methods [2]. The primary strategy involves the surgical resection of tumors, complemented or preceded by chemotherapy and localized radiotherapy. However, when tumors are non-resectable or have metastasized, chemotherapy remains the only therapeutic option to control the spread and size of the cancer.

Chemotherapy, while fundamental, possesses several drawbacks. The non-specificity of cytotoxic agents, which fail to target only cancer cells, leads to systemic side effects, severely limiting the dosages that can be safely administered. In addition, hydrophobic chemotherapeutic drugs exhibit poor solubility and permeability, which can further reduce treatment efficacy [3]. These limitations reduce the therapeutic value of many anticancer drugs and their respective chemotherapy treatments.

Within this context, nanoparticles (NPs) have emerged as a promising tool. They offer enhanced drug delivery capabilities that improve the pharmacokinetics and safety profiles of anticancer treatments. NPs facilitate targeted drug delivery through both passive and active mechanisms, significantly reducing the side effects associated with conventional chemotherapy [4]. Additionally, NPs can be prepared with diverse physicochemical and surface properties that can be tailored to enhance cellular delivery, increase circulation times, and control cargo release [4]. Some nanomaterials are designed with inherent optical or chemical properties that enable stimuli-responsive therapy. Therefore, NPs can be “smartly designed” for different applications such as enhanced drug delivery, phototherapy, vaccination, immunotherapy, imaging, or combination therapies [5].

Despite these advantages, efficient biointerfacing of nanosystems with biological systems remains a significant challenge. Once NPs enter the bloodstream, they encounter a complex environment designed to recognize and eliminate foreign entities. This recognition limits the NPs’ success due to low circulation time, early clearance from the body, and unwanted immune responses [6]. This causes the clinical approval rate of cancer nanotherapeutics to be low [7]. These shortcomings emphasize the need for continued innovation to better disguise nanovehicles from the immune system, prolong circulation time, and enhance targeting capabilities.

Bottom-up fabrication strategies, where ligands and polymers are attached onto the surface of NPs to achieve these goals, still struggle to replicate the multifactorial properties of biological systems and achieve efficient biointerfacing. First reported in 2011 by Liangfang Zhang’s lab, cell membrane (CM)-coating nanotechnology emerged as a top-down biotechnology to produce stealth biomimetic nanosystems [8]. In this method, the CMs from cells are isolated and transferred onto the surface of NPs, resulting in a CMs-coated NPs (CMs-NPs) that inherits the proteins, carbohydrates, and lipids from the source cell [9], [10]. In this initial study, red blood cells (RBCs) membranes provided “stealth” properties to polymeric NPs, with RBCs-coated NPs showing a circulation half-life substantially longer than PEGylated NPs. Subsequent research by the same group explored coating NPs with tumor cells to enhance tumor-targeting capabilities and their use in vaccination [11], as well as the development of platelets (PLTs) CMs-NPs and their biointerfacing properties [12]. Similarly, Parodi et al. in 2013, were among the first to develop a biomimetic system using leukocyte CMs coated on silicon particles [13].

Nowadays, CMs-NPs are celebrated for their ability to merge the synthetic advantages of NPs with the biological benefits of natural CMs [14]. These benefits include enhanced biodistribution, reduced immunogenicity, and the ability to specifically target diseased cells through receptor-mediated interactions [9]. Membrane coating technology has been applied to many cell types, including PLTs, leukocytes, cancer cells, and stem cells, among others. The versatility of this approach has been demonstrated across various applications, showing interesting properties and significant impacts in different pathologies. Notably, Lianfang Zhang’s lab and Cellics Therapeutics, Inc. received approval to proceed with clinical trials for their lead drug product, CTI-005, Human RBCs-Nanosponges, for patients hospitalized with methicillin-resistant *Staphylococcus aureus* or methicillin-sensitive *Staphylococcus aureus* pneumonia [15], [16]. Similarly, the Macrophage Nanosponge (Mϕ-NS) Cellics’ CTI-111, has received funding to advance its application for the treatment of sepsis and other conditions with unmet medical needs such as post-CAR-T cytokine release syndrome (CRS) [17]. This technology have also shown promise in treatments for COVID-19, further demonstrating the versatility of this invention [18]. These advancements underscore the continuous growth and potential importance of CM-nanotechnology in diverse applications.

In this review, we focus on the application of this technology to cancer management. We describe the preparation of CMs-NPs, the diverse cell sources used, and the various treatments these nanosystems can facilitate. Additionally, we offer a forward-looking perspective on the challenges that must be addressed as this technology transitions from the laboratory to clinical settings.

## 2 Description of the technology

**Fig. 1:**
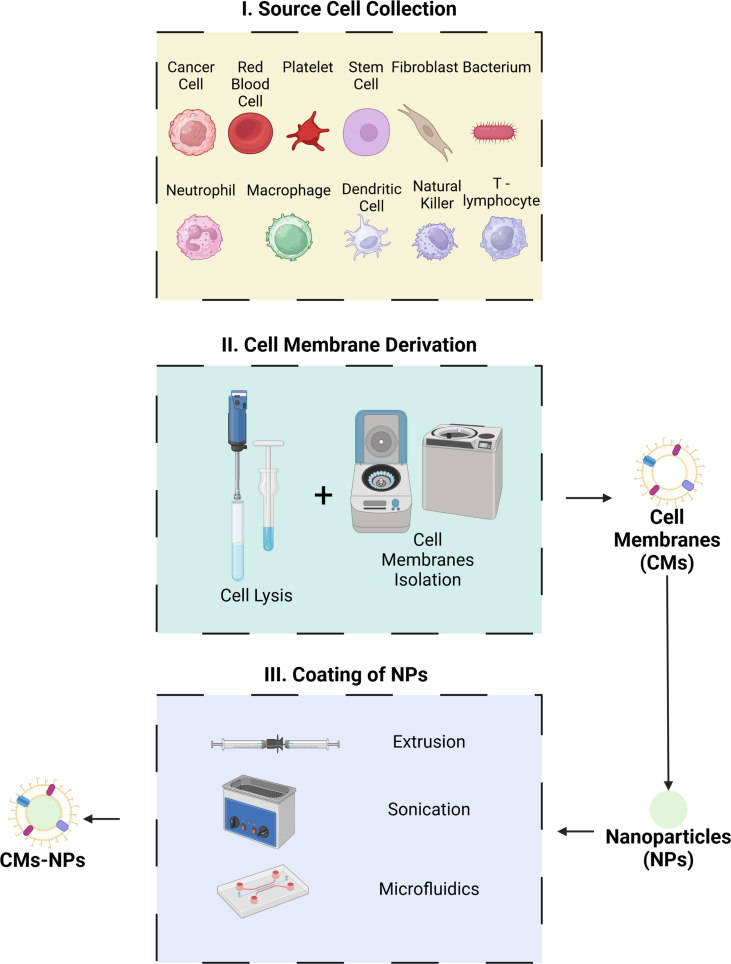
Overview of methodologies to prepare CMs-NPs. The first step involves the production and collection of cells. Next, cell lysis is performed to extract the CMs, which are then isolated using centrifugation techniques. The obtained CMs are subsequently used to coat NPs through various methods, including extrusion, sonication, and microfluidics, to produce CMs-NPs. Created with www.BioRender.com.

To produce CMs-NPs, two main steps are necessary. The first involves the derivation of CMs, which includes the lysis and fragmentation of cells and the isolation of their membrane fragments. The second step is the coating of these membranes onto the chosen nanocarriers. A variety of cell sources have been utilized, including RBCs, PLTs, stem cells, leukocytes, cancer cells, fibroblasts, and bacteria, among others (Fig. 1). The selection of the cell source is crucial and greatly influences the application of the biomimetic nanosystem, which is further explored in Section 3.

### 2.1 Cell membranes derivation

#### 2.1.1 Cell lysis

The preparation of CMs-NPs starts with the extraction of CM material from an appropriate source. For primary blood cells like RBCs and PLTs, the availability of established blood collection and processing infrastructures facilitates their procurement from commercial entities. For nucleated cells, cell cultures on a moderate scale within a laboratory setting typically meet the requirements for preclinical investigations. Notably, suspension cells are amenable to volumetric growth in shaker or spinner flasks, streamlining their collection compared to adherent cells, which need to be detached either mechanically or enzymatically.

The next step is the extraction of the membrane material. This typically involves employing a hypotonic lysis buffer [8], [11], [19], [20], [21], [22], [23], [24], [25], [26]. Different hypotonic buffer compositions are used, with Tris–HCl supplemented with protease inhibitors being the most commonly employed. However, hypotonic PBS [19] and commercial buffers like RIPA are also used [27].

The following step is the homogenization of the cells. Mechanical disruption is typically employed, as it avoids chemical alternatives that may damage the CM structure. This mechanical homogenization involves the use of a Dounce homogenizer or automatic homogenizers [25], [28], [29]. This procedure is performed on ice to avoid sample degradation. Sonication treatment is also employed, which may involve the use of a bath sonicator [30], [31], [32], [33] or an ultrasonic probe or disruptor [34], [35], [36], [37]. The freeze-thaw method is also a well-established technique consisting of subjecting cells to multiple cycles of freezing, either in liquid nitrogen or at −80 °C, followed by thawing at 37 °C. [38], [39], [40]. Nitrogen cavitation, where nitrogen bubbles are formed inside a cell to break them, is also used [41], [42]. Interestingly, this last method, although not often used, was early reported in 1980 by Klempner et al. to isolate plasma membranes of neutrophils [43].

#### 2.1.2 Cell membranes isolation

After cell lysis, the resulting homogenate undergoes differential or gradient centrifugation to isolate plasma membranes. For anucleated cells, this can easily be done by high-speed centrifugation in order to form a pellet containing the CMs [35], [44], [45]. The process is more complex in the case of nucleated cells, requiring the separation of the plasma membrane from intracellular organelles and proteins. Typically, a two-step process is adopted. In the initial centrifugation phase, the mixture is subjected to a lower g-force centrifugation, approximately 600–3000 × g, to predominantly sediment nuclei and non-lysed cells. The post-nuclear supernatant (PNS) is then collected for further processing. To enhance membrane recovery, the pellet is often resuspended in fresh hypotonic buffer and homogenized again. Subsequent centrifugation steps, one or two in number, are applied to the collected supernatant to precipitate the membrane fragments. Variability exists in subsequent procedures, with some protocols centrifuging from 14,000 to 20,000 × g for 20–30 min, using the resulting pellet as isolated CMs [27], [46], [47], [48], while other reports discarding this pellet and further ultracentrifuging the remaining supernatant at 80,000–150,000 × g for 30–60 min [11], [23], [26], [37], [49], [50]. Sucrose gradient centrifugation is also utilized, where the PNS is layered onto a discontinuous sucrose gradient (55–40%–30% w/v sucrose) and centrifuged at 28,000 × g for 30–45 min. The fraction at the 30%–40% interface is collected, washed, and further centrifuged to obtain isolated CMs [44], [51]. As observed, there is a lack of consensus on centrifugation protocols, which are highly dependent on the cell type and the laboratory infrastructure. In this context, we evaluated and compared various centrifugation techniques to elucidate their effectiveness for cancer CMs isolation, showing that the sucrose gradient protocols achieved the purest cell membranes [52]. Similar results has recently been reported [42]. Furthermore, there are different groups that employ membrane protein isolation or cell fractionation kits to derive their CMs [53], [54]. Interestingly, Liu et al. compared a membrane protein extraction kit with a Dounce homogenizer extraction [55]. Lipidomics analysis revealed that the lipid composition of the CMs from the two methods was similar.

Following CMs isolation, a washing step with PBS or another suitable buffer is typically performed, after which the CMs are usually preserved at −80 or −20 °C or lyophilized.

### 2.2 Coating NPs with cell membranes

#### 2.2.1 Nanoparticles

The selection of NPs as cores for CMs-NPs is primarily influenced by the intended application of the final system, as the core material affects the therapeutic modality [56]. Various NPs have been utilized for cancer therapy, including liposomes [57], [58], [59], protein-based NPs [60] or silica NPs [61], [62]. PLGA NPs, favored for their biodegradability and versatility in encapsulating a wide range of substances, are currently the most studied core in this technology [50], [63], [64], [65]. Metallic NPs are also utilized for their imaging and photothermal properties, including iron-based NPs [45], [66], gold-based nanosystems [67], [68] or Metal-Organic Frameworks (MOFs) [38], [49], [69]. Upconversion NPs, capable of converting near-infrared radiation into visible radiation, have also been investigated for their potential applications [70], [71].

As described, a wide range of core designs can be employed in the production of CMs-NPs, tailored according to their specific uses. Nevertheless, a critical requirement for these NPs is a negative surface charge, which ensures effective CM wrapping around the NP due to electrostatic repulsion with the negatively charged CMs. Unlike negatively charged NPs, which can be extruded easily, it has been reported that mixtures of positively charged NPs and negatively charged membranes typically result in significant aggregation [21], [72].

#### 2.2.2 Coating procedures

Various methods are utilized to coat NPs with CMs, with extrusion and sonication being the most common techniques. The initial approach, pioneered by Lianfang Zhang’s lab in 2011, utilized extrusion, which involves passing CMs and NPs cores through polycarbonate membranes with progressively smaller pore sizes, ranging from 800 nm to 200 and 100 nm [8]. This method reshapes the CM, allowing it to envelop the NPs, forming a core–shell structure. The success of CMs-coating *via* extrusion is influenced by the CMs-to-NPs ratio and the surface charge of the NP core, which affect the extent of membrane coverage [73]. The process typically involves multiple cycles of extrusion to ensure complete coating [20], [74], [75]. A major limitation of this method for large-scale production is the significant loss of material due to accumulation on the porous membrane [76].

Alternatively, sonication employs ultrasonic energy to facilitate the coating process, which is less labor-intensive and more suitable for small-scale production. In this method, the CM is reconfigured around the NPs serving as cores through noncovalent interactions. Unlike extrusion, sonication prevents material loss during the coating process. Sonication can be performed using either a bath sonicator [58], [77] or an ultrasonicator [38], [49], [63]. However, despite its widespread use, there is limited data on how sonication parameters affect coating efficiency, often resulting in variable membrane coverage across different studies. Within this context, Yang et al. conducted systematic studies to optimize sonication parameters like amplitude, duration, temperature, and sample volume to enhance the reproducibility of membrane coating [78]. They reported that sonication at controlled lower temperatures and precise amplitude settings helps maintain the integrity and uniform coating of the NPs. These factors must be carefully adjusted to avoid aggregation and achieve consistent results across batches [78]. However, ultrasounds exposure can alter the NPs acting as cores due to cavitation effects [79].

Combining sonication with extrusion is often employed to enhance the coating efficiency, typically starting with a sonication treatment followed by an extrusion procedure to ensure thorough and uniform NP coverage, thus trying to avoid the drawbacks of each of the methods [80], [81]. Although sonication and extrusion are the most employed methods, due to the importance of this step in CM-coating technologies, other methods have also been explored. Rao et al. integrated microfluidics with electroporation to enhance precision and scalability in the preparation of CMs-NPs [82]. The reported device consists of a Y-shaped merging channel, an S-shaped mixing channel, and an electroporation zone right before the outlet. As this mixture moves through the electroporation zone, electrical pulses facilitate the incorporation of magnetic NPs into the RBCs-vesicles by generating temporary hydrophilic holes through the CMs using quick high-voltage electric field pulses, resulting in the formation of RBCs-NPs. Compared to RBCs-NPs created by conventional extrusion, those produced *via* microfluidic electroporation exhibited superior colloidal stability and enhanced performance *in vivo*. Similarly, Han et al. employed microfluidics combined with sonication. This method utilizes microfluidic sonication to exert disruptive forces that dismantle the natural CM structure, and then reassembles it around PLGA cores. These nanosystems are produced in a single step, enhancing both scalability and precision. The microfluidic approach not only simplifies the production of CMs-NPs but also shows promise for large-scale manufacturing and clinical applications due to its efficiency, reproducibility, and storage capabilities [82], [83].

After the coating process, the isolation of CMs-NPs typically involves centrifugation, utilizing the density differences between the NPs core and the CM. Techniques for assessing the coating’s completeness and integrity include transmission electron microscopy (TEM), dynamic light scattering (DLS), ζ-potential evaluation, gel electrophoresis, and colloidal stability tests.

#### 2.2.3 Advancements in coating techniques

The effective coating of NPs is the crucial step in this nanotechnology. The integrity and efficiency of these coatings significantly influence the performance of such systems. Currently, extrusion and sonication are the primary methods employed for applying these coatings, with supplementary techniques from emerging microfluidics. Despite advancements, achieving a perfect coating remains a challenge, continually driving efforts to optimize these processes. Traditional methods used to characterize these systems are generally qualitative and insufficient to assess the degree and variability of the coating in a statistically meaningful way.

To address this, the laboratory led by Vesa-Pekka Lehto introduced a fluorescence quenching assay to quantify fully coated NPs, marking a substantial advance in coating assessment techniques [21]. They observed that following standard protocols – sonication and extrusion – over 60% of silica NPs were inadequately coated, indicating significant potential for protocol refinement. Further explorations by Zou et al. have investigated how NPs elasticity influences the formation of CMs-NPs and their interactions at the nano-bio interface [84]. They reported that softer NPs facilitated a more protein-rich membrane coating, thus providing deeper insights into how NPs properties and membrane coatings may be optimized.

Further, Lehto’s team innovated a method to enhance coating efficiency by introducing external and synthetic phospholipids to augment membrane fluidity, promoting a complete fusion with the core NP [55]. The resulting Hybrid Silica NPs demonstrate a higher full coating ratio (∼23%) and improved tumor-targeting capabilities compared to those coated by traditional methods (full coating ratio of ∼6%) [55]. Additionally, this method reduces the necessary amount of CMs needed, which could be a crucial step in the scaling and translatability of this technology. Hybrid CMs-coated NPs (HCMs-NPs) could also benefit from this intermediate platform, as the inclusion of different CMs types can be achieved using a simpler method compared to current CM-fusing technologies (see Section 3.8). The integration of hybrid CMs and liposomes has also been explored in other works [19].

Moreover, to optimize CM-coating on non-spherical NPs, a novel strategy involving polyethylene glycol (PEG)-assisted coating was developed [85]. In this technique, PEG molecules chemically functionalize the surface, coupled with an innovative solvent-drying technique to ensure successful cancer CMs coating, demonstrating potential for enhanced applications in irregularly shaped NPs [85].

Therefore, CMs coating nanotechnology continues to evolve, with the coating process itself being a primary focus due to its significance in the resultant biointeractions and performance of CMs-NPs. These developments provide crucial insights into the design of functional and active tumor-targeting delivery systems, with implications for both *in vitro* and *in vivo* studies.

**Fig. 2:**
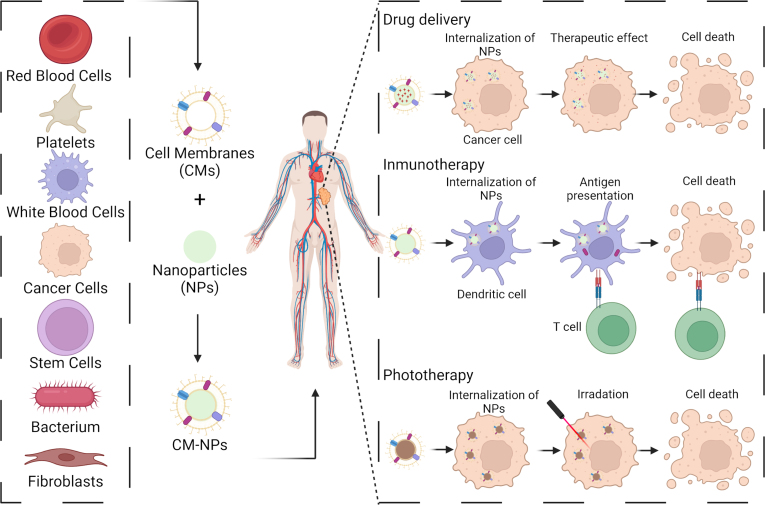
Applications of CM-NPs derived from various cell sources for tumor treatment, including targeted drug delivery, immunotherapy, and phototherapy. In drug delivery, the biomimetic properties of the derived CMs enable long-circulating and targeted CM-NPs, enhancing therapeutic efficacy while minimizing adverse effects. For immunotherapy applications, CM-NPs can function as antigen sources, deliver immunostimulatory agents, or interact with immune cells to enhance antitumor immunity. The illustration depicts their ability to transport tumor-associated antigens to dendritic cells, facilitating antigen presentation to T cells and triggering a targeted immune response against cancer cells. For phototherapy, CM-NPs can act as carriers for photothermal and photosensitizing agents or serve as therapeutic agents due to the properties of their core. Upon irradiation, these nanoparticles exert therapeutic effects while minimizing damage to surrounding healthy tissues. Created with www.BioRender.com.

## 3 Targeting tumors using CMs-NPs

CMs-coated platforms are increasingly studied for their anticancer applications due to their distinctive biointerfacing characteristics [10]. The inherent biological and immunological compatibilities of CMs-NPs offer a unique advantage in this context. Initially, they were engineered with RBCs coatings in 2011, demonstrating a prolonged circulation half-life compared to PEGylated NPs [86]. The use of various cell sources has demonstrated the targeting capabilities of these CMs-NPs, enhancing the delivery of various therapeutic agents. This makes CMs-NPs valuable in a range of treatment modalities, including drug delivery, phototherapy, and immunotherapy [10] (Fig. 2).

The use of CMs-NPs in phototherapy leverages their targeting capabilities to administer photosensitizers and other therapeutic agents for photothermal therapy (PTT) and photodynamic therapy (PDT). PTT converts light to heat *via* NPs, causing localized cellular damage, while PDT uses photosensitizers to generate reactive oxygen species (ROS), enabling targeted cell destruction [56]. Both PTT and PDT are vital components of nanomedicine. CMs-NPs have proven effective as carriers, enhancing the efficacy of these therapies.

In the realm of immunotherapy, there is growing interest in harnessing the immune system to eradicate tumors [87]. Various immunotherapeutic approaches, such as cancer vaccines, adoptive cellular immunotherapy, cytokine immunotherapy, and immune checkpoint blockade inhibitors, are under investigation in clinical settings. CMs-NPs play a pivotal role in this research, delivering immunostimulatory signals and modulating the immune response, demonstrating their potential in advancing cancer immunotherapy and the development of nanovaccines [10], [87].

Applications of CMs-NPs are highly related to the cell source employed to obtain the CMs. The characteristics of CMs-NPs are significantly influenced by the origin of their CM, which can be tailored for specific uses in cancer treatment. The choice of cell type is determined by the target tissue or the intended application. Often, cancer cells are utilized to target the matching cancerous tissue specifically, whereas white or red blood cells are selected for broader targeting purposes. These cell types are predominantly used to enhance the targeted delivery of NPs to designated tissues. Additionally, certain cells are utilized not only for targeting but also to stimulate an immune response against cancer, such as bacterial cells [10].

### 3.1 Cancer cells

Although RBCs were the first cell source used [86], cancer cell coatings have become one of the most significant and commonly employed sources due to the tropism of cancer cells for tumor tissues, as well as their proliferative capacity and ease of culture. When NPs are coated with cancer CMs, they inherit the membrane’s properties, including their propensity for mutual adhesion and the ability to evade the innate immune system—factors that are crucial for tumor growth and metastasis. These characteristics improve the pharmacokinetics and targeting abilities of CMs-NPs. Moreover, cancer CMs are a rich source of tumor-associated antigens and neoantigens, which are beneficial for the development of cancer vaccines and related therapeutic strategies [87]. CMs-NPs constitute an ideal nanovaccine platform where the cancer membrane coating, combined with an immunostimulatory NP core, can induce a potent antitumor immune response. This dual functionality of tumor targeting and immunotherapy was first reported in 2014 [11]. Following this landmark study, tumoral CMs have been extensively utilized for cancer treatment (Table 1).

Scully et al. developed PLGA NPs coated with cancer CMs derived from 4T1 murine mammary cancer cells to specifically target and treat triple-negative breast cancer (TNBC). These NPs encapsulated ABT-737, a *Bcl-2* inhibitor, leveraging the potential of *Bcl-2* inhibition as a promising strategy for treating this aggressive disease. The use of 4T1 cancer CMs significantly enhanced the NPs’ targeting capabilities, allowing for increased homing and accumulation at tumor sites, thereby improving the delivery efficacy of ABT-737 [90]. Harris et al. developed a targeted chemotherapy approach for acute myeloid leukemia (AML) by coating polymeric NPs encapsulating doxorubicin (DOX) with membranes derived from human AML cells (CHRF-288-11 cell line). The study demonstrates that these CMs-NPs induce up to 80% apoptosis in targeted cells, a significant improvement over traditional DOX delivery methods [50].

**Table 1:** NPs coated with Cancer-CMs for tumor management. EPI: Epirrubicin, DEX: dexamethasone, DOX: Doxorrubicin, Gox: Glucose oxidase, GPNA: glutamine transporter antagonist l-γ-glutamyl-p-nitroanilide, TLR7: Toll-like receptor. When the targeted organ is not specified, it corresponds to the tumor itself.

Cell type	NP	Cargo	Notes	Application	Disease/ Targeted organ	Reference
Breast Cancer	ZIF-8 NPs	EPI, Gox and hemin		Immunotherapy	Breast cancer	[37]

Cervical Cancer	PLGA NPs	DEX		Chemotherapy	Cervical cancer	[20]

Cervical Cancer	Silica NPs	siRNA and DOX		Chemotherapy	Cervical cancer	[22]

Melanoma	PLGA NPs		Engeniered cells expressing Ovoalbumin and CD80	Immunotherapy	Melanoma/ Lymph nodes	[88]

Melanoma	PLGA NPs	Photosensitizer and TLR7		Photo- and Immunotherapy	Melanoma	[24]

Melanoma	PLGA NPs	TLR7, imiquimod, and mannose		Immunotherapy	Melanoma	[89]

Colon cancer	MOFs	siRNA		Photo- and Immunotherapy	Colon cancer	[38]

Breast and Lung cancer	CuPt NPs	Cisplatin		Chemotherapy	Lung cancer	[31]

Squamous cell carcinoma	MOFs			Thermo- and Immunotherapy	Squamous cell carcinoma	[49]

Breast Cancer	Iridium Oxide NPs	GPNA		Chemotherapy	Breast cancer	[47]

Breast cancer	PLGA NPs	ABT-737		Chemotherapy	Breast cancer	[90]

Acute Myeloid Leukemia	Polymeric NPs	DOX		Chemotherapy	Acute Myeloid Leukemia	[50]

Multiple myeloma	Polymeric NPs	Bortezomib		Chemotherapy	Multiple myeloma	[23]

Surgically removed tumor	Quantum dots		NPs loaded into a hydrogel	Photo- and Immunotherapy	Breast cancer	[91]

Surgically removed tumor	Polymeric NPs	Imiquimod		Immunotherapy	Prostate cancer / Lymph nodes	[92]

**Fig. 3:**
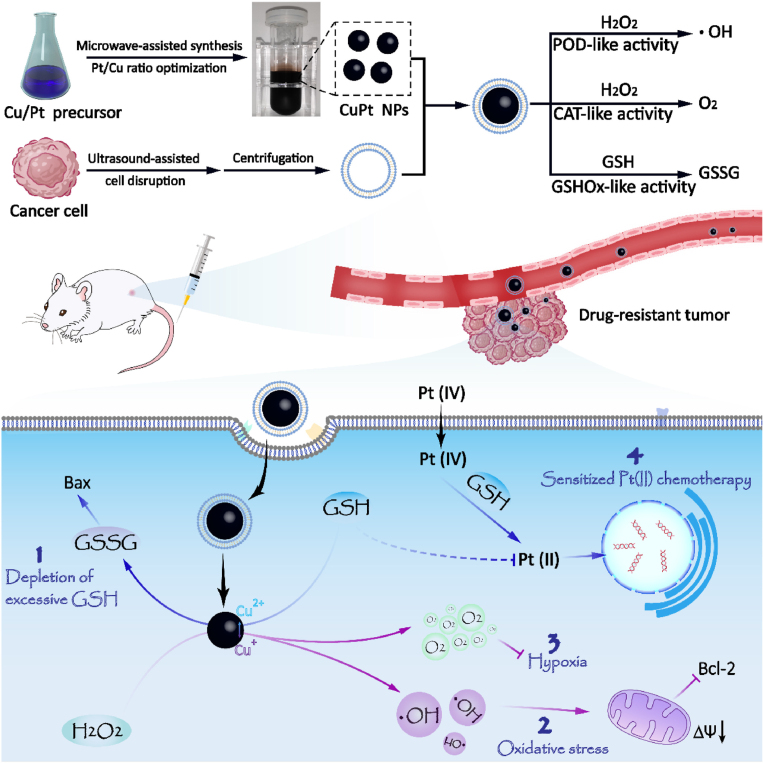
Preparation and fabrication of CuPt NPs coated with cancer CMs (mCuPt) and their synergistic antineoplastic effects. mCuPt modulates the TME by catalyzing the conversion of endogenous H_2_O_2_, and depleting intracellular GSH. This dual action relieves tumor hypoxia and increases oxidative stress. The resulting excessive ROS induces mitochondrial dysfunction, reducing cellular ATP supply and decreasing cisplatin excretion, ultimately reversing cisplatin resistance.

Targeting the TME is a valuable strategy since the TME protects and supports the tumor, often leading to chemotherapy failure. In this sense, Gan et al. introduced a novel CuPt NPs coated with cancer CMs (mCuPts), designed to enhance the efficacy of cisplatin prodrugs and combat chemoresistance in tumors. These mCuPts actively target tumor sites through homotypic targeting, disrupt redox balance, deplete GSH, and relieve hypoxia in cancer cells, effectively modulating the TME (Fig. 3). These modifications of the TME make the tumor more susceptible to Pt(IV) and help overcome cisplatin resistance. Further combination of mCuPts with cisplatin effectively reverses tumor cisplatin resistance and suppresses tumor growth in cisplatin-resistant lung cancer models [31]. Similarly, Li et al. developed dexamethasone-loaded PLGA NPs coated with ovarian cancer CMs [20]. This nanoplatform significantly remodeled the TME, improving the penetration of Doxil and substantially enhancing its therapeutic efficacy against cervical cancers. Ren et al. introduced iridium oxide NPs coated with 4T1 CMs and loaded with the glutamine transporter antagonist l-γ-glutamyl-p-nitroanilide. These NPs exploit iridium oxide’s catalytic properties to alter the TME, enhancing oxygen generation, reducing hypoxia, and increasing oxidative stress, thereby blocking key metabolic pathways [47].

**Fig. 4:**
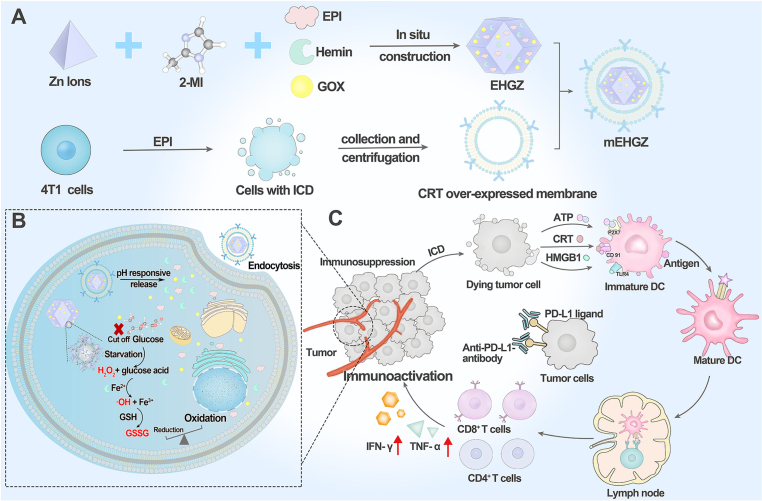
Development of CMs-NPs to induce immunogenic cell death and activate an immune microenvironment, thereby enhancing the therapeutic effect of anti-PD-L1 antibodies. (A) Preparation of mEHGZ system by encapsulating epirubicin, glucose oxidase and hemin in ZIF-8 NPs and coating the NPs with the calreticulin over-expressed 4T1 tumor CMs. (B) Effect of the released epirubicin after mEHGZ internalization in response to low pH kills tumor cells in an immunogenic way. Glucose oxidase and hemin mediate a cascade reaction for generation of ROS and depletion of intracellular GSH strengthening the immunogenic cell death effect. (C) The released antigens after immunogenic cell death promote DCs maturation, CD8^+^T cells infiltration and cytokines secretion to create an supportive microenvironment to boost the therapeutic effect of anti-PD-L1 treatment.

Cancer cell coatings are showing significant promise in immunotherapy. Z. Li et al. developed ZIF-8 NPs encapsulating epirubicin, glucose oxidase, and hemin, coated with a calreticulin-overexpressed 4T1 CMs to improve the response rate of immune checkpoint inhibitors such as anti-PD-L1 in TNBC (Fig. 4). epirubicin serves as an inducer of immunogenic cell death, while glucose oxidase and hemin mediate the cascade generation of ROS to amplify this immunogenic cell death effect in tumor cells. The calreticulin-rich membrane acts as an “eat me” signal, promoting the presentation of released antigens by dendritic cells (DCs). This process enhances the tumor-immunity cycle, resulting in a robust maturation of DCs and a CD8^+^ T cell response, thereby creating an immune-activated microenvironment that makes tumor cells more susceptible to anti-PD-L1 therapy [37]. In another approach, Yang et al. prepared a vaccine using PLGA NPs coated with melanoma CMs, including a toll-like receptor 7 agonist, imiquimod, and mannose to aid antigen-presenting cell recognition. This nanovaccine is efficiently taken up by dendritic cells, inducing maturation and activating antitumor immunity, especially when combined with checkpoint-blockade therapy, resulting in significant treatment efficacy against established tumors [89].

[31].

Combination of immunotherapy and phototherapy is often used. For instance, Chen et al. developed PLGA NPs loaded with tetrakis(4-carboxyphenyl)porphyrin as a photosensitizer and a Toll-like receptor-7 agonist as an immune adjuvant, enveloped by a cancer CM that acts as a tumor antigen. These CMs-NPs effectively kill tumor cells through PDT while simultaneously triggering robust host antitumor immune responses [24]. In another approach, Bai et al. prepared MOFs coated with tumoral CMs for the targeted administration of a photosensitizer and small interfering RNA that targets cyclin-dependent kinase 4. This nanoplatform halts the tumor cell cycle by inhibiting cyclin-dependent kinase 4, but also uses PDT, activated by laser irradiation, to facilitate the release of tumor antigens and attract dendritic cells [38]. Similarly, Cui and colleagues engineered a multifunctional nanosystem utilizing gadolinium-based MOFs loaded with a PD-1 inhibitor and coated with SCC7 cancer CMs, aimed at enhancing magnetic resonance imaging guided cancer thermotherapy and synergistic immunotherapy. The design allows the NPs to effectively target tumor tissues, leverage microwave responsiveness to induce immunogenic cell death, and enhance T-cell activity in the TME [49].

[37].

**Fig. 5:**
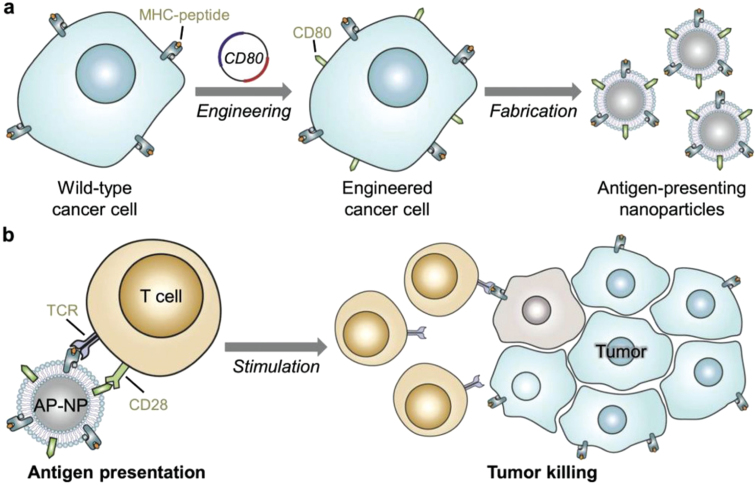
Schematic of engineered CMs-NPs for direct antigen presentation. (A) The wild-type B16-F10 murine melanoma cell line, which naturally present their own antigens *via* MHC-I, are engineered to express CD80. The CMs from these cells are then derived and coated onto PLGA NPs. (B) The resulting NPs with antigen-presenting capabilities can stimulate tumor antigen-specific T cells through T cell receptor and CD28. After activation, T cells are capable of killing cancer cells that express the same antigens.

While natural membrane coatings might express the ligand, such expression might not be at a density that is sufficient to facilitate a strong targeting effect. To address this limitation, various methods have been employed to enhance the functionality of CMs-NPs. One such method involves genetic engineering to alter CM protein expression [93]. This innovation is primarily implemented in cancer culture models due to their ease of use. Jiang et al. demonstrated this approach by developing PLGA NPs coated with cancer CMs genetically modified to express the co-stimulatory molecule CD80 (Fig. 5). These CMs-NPs ensure robust presentation of both the tumor antigen and CD80, which is crucial for eliciting T cell responses against tumors [88]. This approach has also been applied to treat inflammation. In this sense, Park et al. prepared CMs-NPs expressing the very late antigen-4, which targets vascular cell adhesion molecule-1, allowing the delivery of anti-inflammatory drugs, such as dexamethasone, directly to sites of inflammation [29]. Interestingly, this group also developed PLGA NPs coated with membranes expressing the influenza virus hemagglutinin protein, which allows the NPs to effectively escape endosomes, overcoming the cellular endolysosomal pathway that typically degrades foreign materials. These NPs were loaded with a model mRNA to circumvent typical cellular barriers, reaching the cytosol where it is efficiently translated into proteins [28].

Autologous materials derived from a patient’s excised tumor open avenues for personalized therapeutic approaches. Ye et al. developed a personalized photothermal cancer nanovaccine using CMs obtained from surgically removed tumor cells. The resulting CMs-NPs were combined with PD-1 checkpoint blockade antibodies to enhance the immune response against residual and metastatic tumors [91]. Similarly, S. Li et al. developed cancer CMs-NPs derived from surgically excised tumors and loaded with the immune modulator R837. These nanosystems effectively targeted dendritic cells within lymph nodes, enhancing the immune response against prostate cancer. This personalized approach ensures tailored immune activation. Combining these NPs with PD-1 antibodies significantly improved therapeutic outcomes, demonstrating potent anti-tumor efficacy in clinical settings [92].

[88].

### 3.2 Red blood cells

RBCs serve as natural carriers within the body, primarily for oxygen delivery. RBCs are biconcave disks with a diameter of 7–8μm and a central thickness of approximately 1μm [94]. Two major advantages of utilizing RBCs CMs as coatings are their extended circulation life – up to 120 days – and their ability to evade immune detection, facilitated by surface “self-markers” like CD47, which interacts with the mononuclear phagocytic system to prevent immune clearance [95], [96]. In addition, their lack of internal organelles and abundance of hemoglobin simplify the process of membrane purification.

RBCs were the first cell source employed to generate CMs-NPs. In 2011, Zhang et al. demonstrated that PLGA NPs coated with RBCs CMs exhibited longer retention times compared to their PEG-coated counterparts [8]. Since then, numerous studies have validated that RBCs CMs-NPs can significantly enhance the efficacy of disease treatments by prolonging circulation and improving immune evasion (Table 2).

**Fig. 6:**
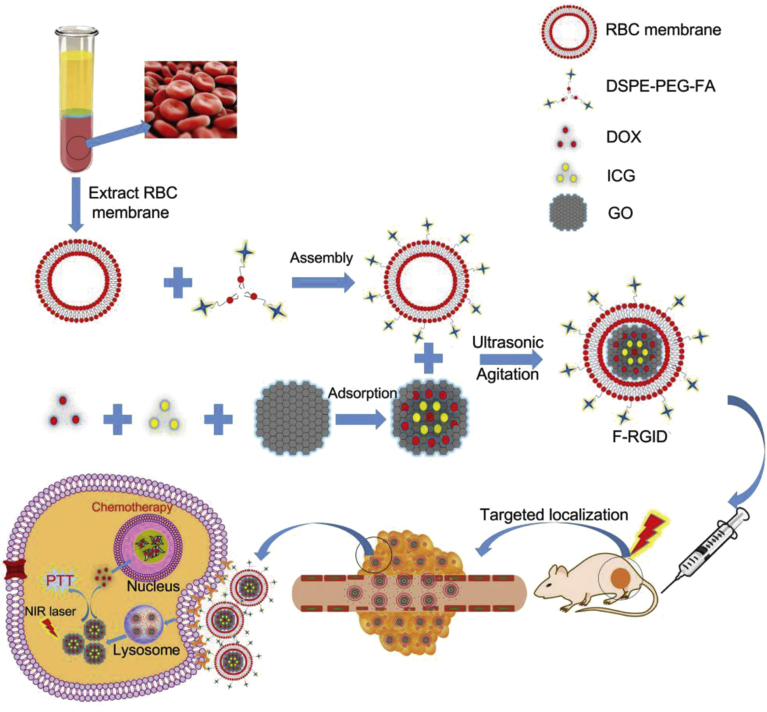
Schematic illustration of F-RGID fabrication and application. This process involves the functionalization of RBCs CMs with FA using a lipid-insertion approach (DSPE-PEG-FA). Graphene oxide-NPs containing DOX and Indocyanine Green (ICG) are further coated using an ultrasonication method. These CMs-NPs are employed *in vivo* for combined chemotherapy and photothermal therapy treatments.

For example, M. Li et al. prepared RBCs-coated upconversion NPs further modified with folic acid (FA) to enhance their targeting abilities to tumor sites. These NPs, which allow for magnetic resonance imaging and upconversion luminescence imaging, showed promising results in early TNBC tumor imaging applications [71]. RBCs-coated NPs are also employed for phototherapy applications. Wang et al. developed a RBCs-coated superparamagnetic Fe_3_O_4_ NPs. These NPs, loaded with near-infrared fluorescence cypate molecules, significantly enhanced tumor targeting, and upon laser irradiation, generated sufficient heat to effectively suppress tumors through photothermal ablation *in vivo* [66]. J. Li et al. engineered graphene oxide-NPs loaded with the photosensitizer Indocyanine Green and the chemotherapeutic agent DOX, thus integrating targeted drug delivery with phototherapy (Fig. 6). The RBCs CMs were further functionalized with the targeting molecule FA *via* a lipid-insertion approach. These CMs-NPs, named F-RGID, demonstrated effective photothermal-chemotherapy in both *in vitro* and *in vivo* studies, showing significant potential in clinical settings [100]. In a combinatorial approach, Rodrigues et al. developed gold-core silica shell nanorods coated with Polyethylenimine and RBCs CMs loaded with acridine orange, facilitating combinatorial chemo-PTT [102].

**Table 2:** NPs coated with RBCs CMs for tumor management. AO: Acridine Orange, CTL: Calcitriol, DiR: 1,1-dioctadecyl-3,3,3,3-tetramethylindotricarbocyanine iodide, DTX: Docetaxel, FA: Folic acid, GO: Graphene Oxide, ICG: Indocyanine Green, PEI: Polyethylenimine, PEG: Poly(ethylene glycol), PTX: Paclitaxel. When the targeted organ is not specified, it corresponds to the tumor itself.

NP	Cargo	Notes	Application	Disease / Targeted organ	Reference
Upconversion NPs		FA functionalization	Imaging	Breast cancer	[71]

Polymeric NPs	PTX and Tetraphenylchlorin		Chemo- and Phototherapy	Cervical carcinoma	[97]

Iron oxide NPs	Cypate		Photothermal therapy	Colon cancer	[66]

Iron oxide NPs			Photothermal therapy	Breast cancer	[98]

PEG Hydrogel NPs	DOX		Chemotherapy	Brest cancer	[99]

GO NPs	ICG and DOX	FA functionalization	Chemo- and Phototherapy	Cervical cancer	[100]

CTL and DTX micelles	DiR, DTX and CTL		Chemo- and Phototherapy	Breast cancer	[35]

β-glucan NPs	DOX and curdlan		Chemo- and Immunotherapy	Melanoma	[101]

Gold-core nanorods	AO	PEI functionalization	Photothermal therapy	Cervical cancer	[102]

[100].

### 3.3 Platelets

PTLs measure 1–3μm in diameter and circulate for about 7–10 days [103]. They are pivotal in maintaining vascular integrity, combating pathogens, and interacting with circulating cancer cells, thus playing roles in immunity, wound healing, and tumor metastasis. PTLs are equipped with surface proteins like p-selectin, CD47, CD55, and CD59, which help them evade phagocytosis, prevent complement system activation, and recognize injured vessels and circulating tumor cells, making them excellent candidates for developing CMs-NPs for applications in oncology, cardiovascular diseases, inflammation, and bacterial infections [104], [105]. However, obtaining, producing, and stabilizing platelet membranes is challenging due to their small size and low blood concentration [103]. Nevertheless, PTLs are one of the most employed cell sources to prepare CMs-NPs, along with RBCs and cancer cells (Table 3).

**Fig. 7:**
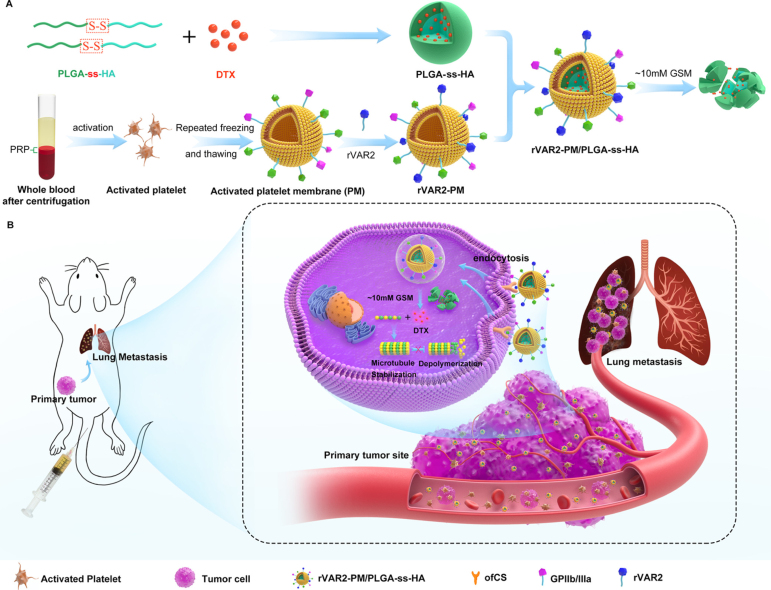
Schematic illustration of (A) the preparation of CMs-NPs by coating responsive PLGA NPs with activated PLTs CMs modified with rVAR2 (rVAR2-PM/PLGA-ss-hemagglutinin), and (B) the targeting of rVAR2-PM/PLGA-ss-hemagglutinin NPs to primary tumors and lung metastatic sites *in vivo*.

As a targeted drug delivery example, Zhou et al. developed redox-responsive CMs-NPs by coating the surface of disulfide-containing PLGA NPs with activated PLTs CMs further modified with the malaria protein VAR2CSA (Fig. 7). These CMs-NPs, containing DTX, employ the tumor-homing capabilities of the activated PLTs CMs, combined with the specific binding affinity of VAR2CSA for oncofetal chondroitin sulfate, which is overexpressed on cancer cells. The study showed that these responsive NPs were endocytosed by cells where they disintegrated, releasing the chemotherapeutic cargo. These nanosystems improved tumor accumulation and enhanced penetration at both primary and metastatic sites, effectively reducing tumor growth and metastasis *in vivo*[65]. Similarly, Zhang et al. employed PLTs CMs to coat mesoporous silica NPs co-loaded with tirapazamine (a hypoxia-activated prodrug) and 5,6-dimethylxanthenone-4-acetic acid (a vasculature-disruptive agent), showing tumor targeting and inhibition [62]. Zhuang et al. employed PLTs CMs for targeted gene silencing *in vivo*. These PLTs-MOFs carrying siRNA targeting survivin demonstrated significant tumor targeting and therapeutic efficacy in a murine breast cancer model [106].

**Table 3:** NPs coated with PLTs CMs for tumor management. DMXAA: 5,6-dimethylxanthenone-4-acetic acid, DTX: Docetaxel, TLR: Toll-like receptor, TPZ: Tirapazamine, HAP: Hypoxia-activated prodrug, PLA: Polylactic acid. When the targeted organ is not specified, it corresponds to the tumor itself.

NP	Cargo	Notes	Disease / Targeted organ	Reference
MOF	siRNA	Chemotherapy	Breast cancer	[106]

PLGA NPs	DTX	Chemotherapy	Melanoma and lung metastases	[65]

Silica NPs	TPZ, HAP and DMXAA	Chemotherapy	Colon cancer	[62]

Iron oxide NPs		Photothermal therapy	Breast cancer	[45]

PLA NPs	TLR agonist	Immunotherapy	Colon cancer, breast cancer and lung metastases	[107]

Silica NPs		CTC-targeting.	Circulating tumor cells	[108]

Silica NPs	Sorafenib and Sora	Chemo- and Immunotherapy	Hepatocellular carcinoma	[61]

PLTs-NPs are also employed for PTT [45] and immunotherapy [107] or combinatorial treatments [61]. Interestingly, J. Li et al. developed silica NPs coated with activated PLTs and functionalized with the tumor-specific, apoptosis-inducing cytokine TRAIL. This design aims to combat metastasis by intercepting circulating tumor cells (CTCs), as PLTs tend to aggregate at sites of injury or tumor interaction, thereby enabling the direct delivery of cytotoxic TRAIL to the CTCs [108].

[65].

### 3.4 White cells

White cells are known to congregate at inflammation sites, which are commonly associated with the onset of cancer. Immune cells are critically involved in the process of cancer development, with certain types either facilitating or hindering tumor growth and metastasis, and are part of the TME [109], [110]. Their capacity to adhere and their membrane-bound active recognition receptors allow them to establish direct contact with tumor cells. These characteristics enable them to traverse biological barriers and home in on specific locations, such as tumor sites, effectively utilizing their evasion mechanisms of the immune system [111]. Various immune cells, such as neutrophils, macrophages, dendritic cells, T cells, and natural killer cells, are employed in creating CMs-NPs (Table 4) [87].

#### 3.4.1 Neutrophils

Neutrophils, which constitute 40 to 70% of all human white blood cells, are vital granulocytes working as the initial line of defense that rapidly mobilize in response to infectious or cancer-related inflammation. Neutrophils possess specific adhesion molecules on their surfaces that enable them to target CTCs and tumor-associated niches. However, their brief half-life of merely 7 h limits their direct application [112]. Xi Cao et al. developed neutrophil-coated PEG-PLGA NPs loaded with celastrol for pancreatic cancer treatment. These nanoplatforms crossed the blood-pancreas barrier, delivering the drug directly to the cancer site, significantly inhibiting tumor growth and prolonging survival in animal models [64]. Neutrophils-NPs have also been employed in malignant glioma [59] and colorectal cancer [113]. For metastasis treatment, Kang et al. developed PLGA NPs loaded with the proteasome inhibitor carfilzomib, utilizing the cancer-targeting properties of these CMs. These NPs showed a significant capacity to deplete CTCs and inhibit the formation of metastases in a mouse model of breast cancer [63].

**Table 4:** NPs coated with CMs from White cells for tumor management. EGFR: Epidermal growth factor receptor, cM70: Pore-forming activity-caged macrolittin, CTCs: Circulating tumor cells, DC: Dendritic cell, DOX: Doxorrubicin, NE: Neutrophil, NK: Natural Killer, RAPA: Rapamycin, TLR: Toll-like receptor, PAS: Proline-alanine-serine, PD1: Programmed cell death protein 1, TCPP: (4,4${}^{\prime }$,4${}^{\prime \prime }$,4${}^{\prime \prime \prime }$-(porphine-5,10,15,20-tetrayl) tetrakis (benzoic acid)). When the targeted organ is not specified, it corresponds to the tumor itself.

Cell type	NP	Cargo	Notes	Application	Disease / Targeted organ	Reference
NE	PLGA NPs	TLR agonist		Immunotherapy	Colon cancer	[113]

NE	PLGA NPs	Carfilzomib		CTCs-targeted therapy	Metastasis (breast cancer)	[63]

NE	PEG-PLGA NPs	Celastrol		Chemotherapy	Pancreatic cancer	[64]

NE	Liposomes	PTX		Chemotherapy	Glioma	[59]

Macrophage	PLGA NPs	Fluorophore	Cells engineered expressing PAS	Prolonged Circulation Time	–	[114]

Macrophage	Upconversion NPs	Rose Bengal		Chemo- and Immunotherapy	Breast cancer	[70]

Macrophage	Liposomes	SN-38		Chemotherapy	Colon cancer	[58]

Macrophage	Curcumine NPs	Curcumine		Chemo- and Photothermal therapy	Colon cancer	[36]

Macrophage	Liposomes	DOX and Tyrosine Phosphatase Inhibitor 1	peptide GPLGIAGQ functionalization	Chemo- and Immunotherapy	Osteosarcoma	[115]

DC	PLGA NPs	IL-2		Immunotherapy	Ovarian cancer	[116]

DC	PLGA NPs	Imiquimod	anti-CD3ϵ functionalization		Melanoma	[117]

DC	PLGA NPs	RAPA		Chemo- and Immunotherapy	Glioma	[27]

NK	PLGA NPs	TCPP		Photo- and Immunotherapy	Breast cancer	[118]

NK	Liposome	DOX		Chemotherapy	Breast cancer	[57]

T-cell	Albumin NPs	ORY-1001	Cells engineered expressing PD1 and cM70	Immunotherapy	Breast cancer	[60]

T-cell	PLGA NPs	Dacarbazine		Immunotherapy	Melanoma and lung cancer	[119]

T-cell	PLGA NPs	DTX and others		Chemotherapy	Breast cancer	[120]

T-cell	Iron oxide NPs		Cells engineered expressing EGFR antibody	Diagnosis	Circulatin tumor cells	[121]

#### 3.4.2 Macrophages

Macrophages play crucial and versatile roles in regulating tissue repair and regeneration upon injury. They are integral to tissue repair, bacterial defense, homeostasis maintenance, and immune responses against pathogens, while also mediating inflammatory reactions [122]. As one of the first lines of defense against infection, macrophages converge on injury sites, where they ingest and decompose cellular remnants and pathogens using toll-like membrane receptors. Additionally, within the TME, macrophages significantly impact tumor development, metastasis, and therapeutic responses. The presence of $\alpha_{4}$ integrin on macrophages enables them to target tumors by recognizing vascular cell adhesion molecule 1 on the surface of cancer cells, improving macrophage-mediated targeting [123]. Furthermore, macrophages are essential for presenting tumor-associated antigens to T cells and for producing cytokines that encourage the growth and anti-tumor actions of T cells and NK cells [124]. These capabilities render macrophage-CMs a promising avenue for NP coating, aiming to prepare biomimetic and targeted CMs-NPs [124]. In addition, macrophages can be easily sourced, cultured, and purified, however, they are a non-replicating population that generally survives between 2–3 weeks under ideal conditions, primarily used in primary culture settings. Macrophage cell lines are also available and are commonly employed.

Combinatorial approaches with macrophage CMs are often used, as this coating enhances biocompatibility, targeting efficacy, and leverages macrophages’ tumor-targeting abilities. Wu et al. prepared macrophage-CMs-coated liposomes encapsulating DOX along with the tyrosine phosphatase inhibitor 1, known for its immune-modulatory effects, for both chemotherapy and immunotherapy of osteosarcoma [115]. In another approach, Chen et al. developed tumor-associated macrophage-CM-coated upconversion conjugated with the Rose Bengal photosensitizer. This strategy inhibits primary breast tumor growth and induces a systemic immune response to address distant metastases [70]. Xi Liu et al. developed a photothermal and chemotherapy approach to treat colorectal cancer. They prepared CMs-NPs composed of curcumin, as the chemotherapeutic agent, modified with polydopamine to enhance photothermal effects [36].

**Fig. 8:**
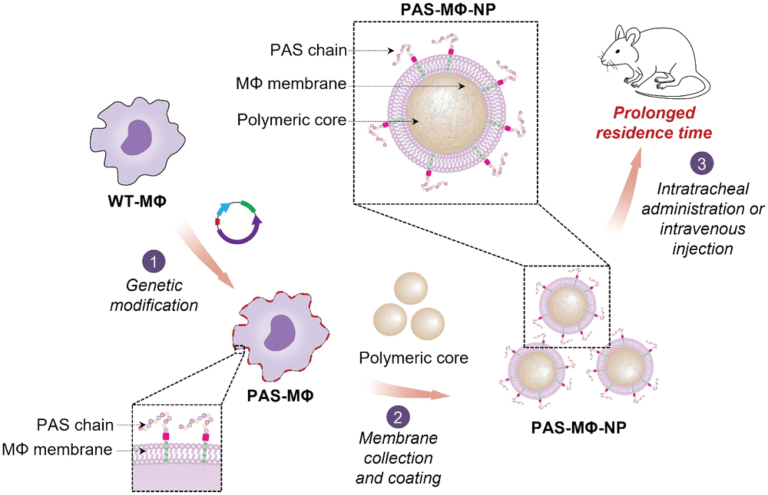
Preparation of CMs-NPs from genetically modified macrophages. Wild-type macrophages are genetically modified to express proline-alanine-serine (PAS) peptide chains on the CMs. The membranes of the PAS-expressing macrophages are then derived and coated onto PLGA NPs. These CMs-NPs exhibit prolonged residence times when injected intravenously or intratracheally into mice.

Macrophages, like cancer cells, can be genetically engineered to express specific molecules. In this regard, Zhang’s lab utilized macrophages genetically engineered to express the proline-alanine-serine peptide to further coat PLGA NPs with PAS-expressing macrophage CMs (Fig. 8). This modification provides additional protection against opsonization, extending the circulation time of the CMs-NPs in the bloodstream [114].

[114].

#### 3.4.3 Dendritic cells

Dendritic cells (DCs) play a crucial role in initiating the body’s targeted anti-tumor immune response. DCs process and present a broad spectrum of antigens, such as nucleic acids, polypeptides, and proteins, to stimulate specific immune responses [125]. Their ability to modulate the immune system offers significant possibilities for treating cancers, autoimmune diseases, and preventing transplant rejections.

**Fig. 9:**
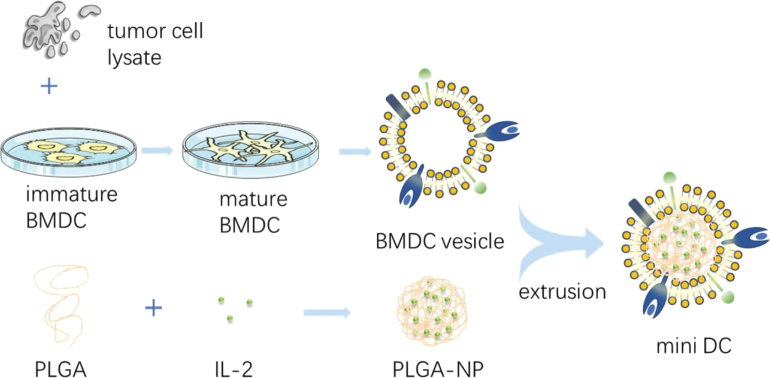
Schematic illustration for the preparation of mini DC: IL-2 loaded PLGA NPs coated with tumor cell lysate-primed DC CMs.

Adapted from: [116].

DC-based vaccines are promising immunotherapies due to their ability to initiate immune responses. Nonetheless, the clinical success of these vaccines is yet to be fully validated. In this sense, Cheng et al. introduced an innovative “miniDC” nanovaccine, composed of PLGA NPs encapsulating IL-2 and coated with CMs from bone marrow–derived dendritic cells primed with ovarian cancer cell lysates (Fig. 9). This design enables the miniDC to exhibit DC-specific plasma membrane proteins such as MHC, CD86, and CD40, effectively mimicking natural DCs’ antigen-presenting functions while concurrently releasing IL-2 to activate T cells and trigger a strong antitumor immune response, presenting an enhanced method for immunotherapy against ovarian cancer [116]. Similarly, Xiao et al. engineered artificial antigen-presenting cells using metabolically labeled DCs incubated with tumor-derived antigens as another immunotherapy approach. These DCs CMs coat PLGA NPs loaded with the immunostimulant imiquimod and further functionalized with anti-CD3ɛ antibodies *via* click chemistry for targeted T-cell activation. The resulting CMs-NP efficiently spreads within lymph nodes, substantially boosting the stimulation of T cells and local antigen-presenting cells [117].

In a combinatorial approach, Xiaoyue Ma et al. developed PLGA NPs containing rapamycin, a chemotherapeutic agent, and coated with CMs from activated DCs primed with tumor antigens for glioblastoma treatment. The prepared CMs-NPs significantly restrained glioma growth and promoted a robust immune response *in vivo* [27].

#### 3.4.4 Natural killers

Natural killer (NK) cells are crucial components of the immune system’s defense against cancer and infectious agents. They possess the ability to recognize and induce apoptosis in target cells through either cytotoxic substance release or receptor interaction [126]. Unlike other immune cells, NK cells can attack tumor cells without prior antigen-specific activation and do not require MHC recognition [126]. Immunotherapy harnessing NK cells is increasingly seen as an effective cancer treatment, complementing traditional therapies [127].

Recent advances include NPs coated with NK CMs (NK-CMs), which have shown great promise for precision tumor therapy. For instance, Deng et al. developed NK-NPs loaded with the photosensitizer TCPP. These CMs-NPs employ the innate immune properties of NK cells, such as targeting and inducing M1 macrophage polarization, to bolster antitumor immunity while the TCPP enhances PDT, killing cancer cells and promoting the release of immune-stimulating molecules [118]. Additionally, Pitchaimani et al. introduced the NKsome, an engineered NK-based liposome with enhanced selectivity for cancer cells. The NKsome, loaded with DOX, displayed significant anti-tumor activity against an MCF-7 induced tumor model *in vivo*, underlining its therapeutic potential [57].

#### 3.4.5 T cells

T cells possess the ability to navigate to tumor sites and identify antigens on the surface of tumors. Equipped with T-cell receptors (TCRs), these cells have a natural affinity for tumors, recognizing and binding to specific molecules on cancer cells. This capability makes TCMs highly effective for targeted tumor therapies [128].

Various NPs coated with T cell-derived CMs have been developed for enhanced delivery of therapeutics. For example, Zhang et al. engineered NPs using crosslinked albumin coated with T lymphocyte membranes expressing programmed cell death protein 1 (PD1). These deliver the epigenetic drug ORY-1001, which induces interferon production and modulates immune responses. They target tumors expressing programmed cell death ligand 1 (PDL1) through PD1/PDL1 interactions, enhancing the activation of immune checkpoint proteins, upregulating intratumoral interferons, and boosting cytotoxic T lymphocyte activity. This method has shown significant antitumor efficacy *in vivo* across various tumor models, including TNBC, melanoma, and colon cancer [60]. In another approach, Kang et al. employed EL4 T-CMs to coat PLGA NPs encapsulating dacarbazine for melanoma treatment. This innovative system leverages the stable expression of various plasma membrane proteins by the EL4 cell line, enhancing the targeting and killing of cancer cells through mechanisms like Fas-ligand-mediated apoptosis and resistance to immunosuppression [119]. We have recently developed PLGA NPs loaded with DTX and coated with CMs from exhausted T-cells. These NPs target tumor cells expressing specific ligands and disrupt the PD1/PDL1 axis, thereby enhancing chemotherapy efficacy. *In vivo* experiments showed that these CMs-NPs accumulate significantly in a PDL1-positive patient-derived xenograft model of TNBC [120]. In an interesting approach, Jiang et al. developed iron oxide NPs coated with genetically modified Jurkat cells as a diagnostic tool for isolating CTCs, which are crucial for early cancer detection and monitoring metastasis. The CMs-NPs surface is coated with Jurkat CMs genetically modified to express a single-chain variable fragment (scFv) of an anti-EGFR antibody, enhancing specificity and binding affinity to EGFR-positive CTCs. This innovative configuration increases binding efficiency by over 100-fold compared to traditional methods and reduces nonspecific interactions with background leukocytes, improving the purity of isolated CTCs [121].

### 3.5 Stem cells

Stem cells, characterized by their unspecialized nature, possess the capabilities of self-renewal and multidirectional differentiation, coupled with low immunogenicity, making them highly beneficial for diverse therapeutic applications. Mesenchymal stem cells (MSCs), a type of pluripotent adult stem cells, can be derived from various tissues, including adipose tissue, peripheral blood, umbilical cord, and placenta [129]. These cells can persist in the human body for extended periods, exhibit tumor specificity, and are readily obtainable with notable immunomodulatory properties and minimal immunogenicity. MSCs demonstrate unique biological attributes and remarkable proliferative capacity *in vitro*, facilitating rapid growth to attain sufficient quantities for *in vivo* treatments [130]. Bioengineering strategies that integrate synthetic NPs with stem cells, leveraging their tumor-targeting and homing capacities, are emerging as promising biomimetic platforms for cancer theranostics (Table 5) [131].

Tian et al. developed MSC-coated PLGA NPs loaded with PTX for targeted breast cancer therapy. These NPs showcased exceptional stability, controlled PTX release, and enhanced antitumor effects *in vitro* and *in vivo* in a 4T1 orthotopic breast cancer model [132]. Similarly, Xie et al. prepared hollow manganese dioxide NPs coated with human umbilical cord MSCs and encapsulating PTX for a targeted drug delivery approach. These CMs-NPs were further functionalized with a TAT peptide for nuclear targeting. The prepared nanosystem, upon intravenous administration, effectively targeted tumor sites, significantly inhibited primary tumor growth, and reduced the toxic side effects of PTX in an orthotopic breast cancer model [133]. Sahar Taghavi et al. developed hollow gold coated with human umbilical cord MSCs and loaded with DOX for both imaging and targeted drug delivery. After administration, these CMs-NPs effectively accumulated at the tumor site, significantly suppressing tumor growth and metastasis in a 4T1 tumor-bearing mouse model [68].

### 3.6 Fibroblasts

Fibroblasts, particularly cancer-associated fibroblasts (CAFs), have gained considerable attention in the context of the TME due to their pivotal role in oncological processes [134]. CAFs contribute to cancer progression by secreting soluble factors and extracellular matrix components, which promote cancer cell proliferation, metastasis, and drug resistance. This activity results in a dense stroma that significantly impedes effective drug delivery [135]. Recent research has underscored the advantages of targeting CAFs to break down this barrier, thereby enhancing the efficacy of cancer treatments. NPs designed to either eliminate CAFs or modify their behavior have demonstrated promising antitumor effects [136]. However, the approach of using fibroblast CMs to coat NPs for cancer theranostics is relatively unexplored (Table 5).

**Fig. 10:**
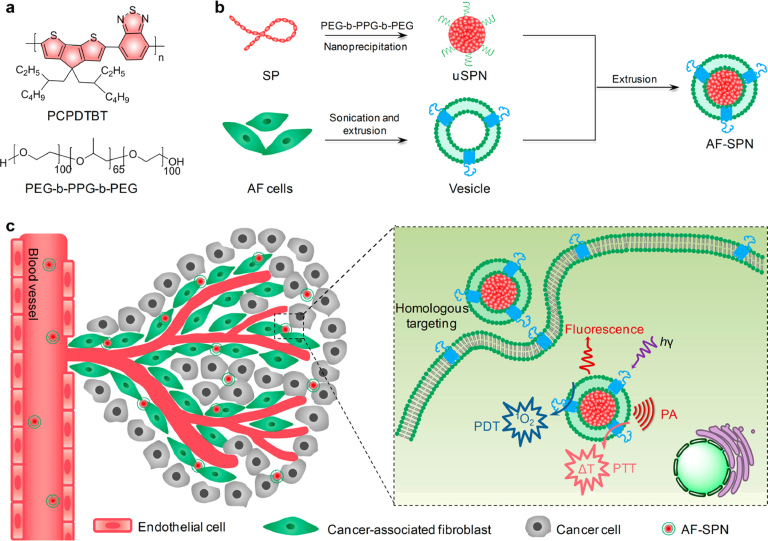
Comprehensive overview of the preparation and application of AF-SPN. (A) Chemical structures of PCPDTBT (semiconducting polymer core) and PEG-b-PPG-b-PEG, which are the key components used in the synthesis of semiconducting polymer (SPNs). (B) Preparation procedure for AF-SPN, demonstrating the sequential processes involved. (C) Homologous targeting mechanism of AF-SPN to cancer-associated fibroblasts (CAFs) within tumor tissues.

A noteworthy study by Li et al. prepared CMs-NPs, namely AF-SPN, by coating a semiconducting polymer core with fibroblast CMs (Fig. 10). These CMs-NPs exhibited preferential targeting of CAFs. This specificity led to increased accumulation in tumor tissue compared to NPs coated with cancer CMs, demonstrating superior phototheranostic capabilities in cancer treatment [137]. Additionally, there is an example of fibroblast-coated NPs used in the treatment of conditions such as rheumatoid arthritis, where fibroblasts are a primary cellular component in the inflamed joints of affected patients [34]. Therefore, fibroblast coatings exhibit significant potential in enhancing the effectiveness of cancer therapies.

[137].

### 3.7 Bacterium

CMs-NPs, derived from bacterial membranes, have been effectively developed. The concept of utilizing bacteria to boost antitumor immunity dates back over a century and remains a vibrant area of research with substantial potential [138]. Bacterial CMs, abundant in immunogenic antigens, are favored for vaccine development due to their crucial role in enhancing adaptive immune responses and activating innate immunity. Particularly, the outer membranes of Gram-negative bacteria are explored for their potential to bolster antitumor immunity [139]. However, the application of bacterial membrane-encapsulated nanomedicines in cancer research remains relatively rare (Table 5).

Zhang’s lab introduced a novel microrobot-based strategy for enhanced cancer immunotherapy, employing a magnesium-based micromotor system loaded with immunogenic bacterial outer membrane vesicles. These microrobots were shown to create large cavities within tumors and promote cell death. In addition, these CMs-NPs stimulated immune cell recruitment and activation, initiating a cascade that significantly suppressed both treated and distant tumor growth upon intratumoral administration [139]. In another approach, Patel et al. developed bacterial CMs-NPs incorporating immune-activating PC7A/CpG multimeric nucleic acids within bacterial membranes for enhanced antigen delivery. These CMs-NPs, combined with radiotherapy and immunotherapy, facilitated the capture of new tumor antigens post-irradiation, increased uptake by DCs, and stimulated the anti-tumor T cell response through cross-presentation [140].

While applications in cancer are still limited, there are developments using CMs-NPs to treat bacterial infections, coated with red blood cells RBCs, macrophages, and neutrophils, among others [67], [141]

**Table 5:** NPs coated with CMs from Stem cells, Fibroblasts and Bacteria, for tumor management. DOX: Doxorrubicin, MSCs: Mesenchymal stem cells, PTX: Paclitaxel. When the targeted organ is not specified, it corresponds to the tumor itself.

Cell type	NP	Cargo	Notes	Application	Disease / Targeted organ	Reference
MSCs	PLGA NPs	PTX		Chemotherapy	Breast cancer	[132]

MSCs	MnO_2_ NPs	PTX	TAT peptide	Chemo- and Immunotherapy	Lung cancer	[133]

MSCs	Gold NPs	DOX		Imaging and Chemotherapy	Metastatic breast cancer	[68]

Fibroblast	Polymeric NPs			Imaging and Phototherapy	Breast cancer	[137]

Bacteria	Polymeric NPs			Immunotherapy	Melanoma and neuroblastoma	[140]

Bacteria	Micromotor (Mg NPs)			Immunotherapy	Colon cancer	[139]

### 3.8 Hybrid cell membranes

The potential of a single CMs coating on NPs has limitations. To enhance NP functionality, a novel approach involves the development of hybrid membrane coatings by merging CMs from different cell types to generate HCMs-NPs. This fusion process combines the surface characteristics of both parent cells. This technique was pioneered by Liangfan Zhang’s lab in 2017, where RBCs-PLTs hybrid CMs were coated onto PLGA NPs [30]. This method enhanced their circulatory longevity and targeting precision, opening new avenues for advanced therapeutic and diagnostic applications [30], [142], [143].

The fusion of different CMs is a process that avoids the need for chemical modification, facilitating mass production. The crucial step in this technology is generating the fused CMs. This can be achieved either by extracting CMs from two different cells and then fusing them or by fusing cells first and then extracting the hybrid CMs [76]. In the aforementioned approach, Zhang’s lab facilitated membrane fusion by stirring two different membranes at 37 °C [30]. Currently, fusing CMs *via* sonication or extrusion, after which the hybrid CMs is coated onto NPs, are the most employed methodologies [144], [145], [146]. Alternatively, a fusion cell is prepared first, usually *via* polymer stimulation or electrofusion. Subsequently, CMs from the fused-cells are extracted using conventional methods and further coated onto NPs. This approach ensures the retention of each cell type’s functional characteristics on the resulting CMs-NPs [147], [148], [149]. The degree to which NPs display specific functions depends largely on the ratio of the combined CMs. The choice of cell source is therefore critical for the specific therapeutic goals of the cancer treatment. Although most studies employ a two CMs strategy for HCMs-NPs, combining more types of membranes has also been explored [144] (Table 6).

**Fig. 11:**
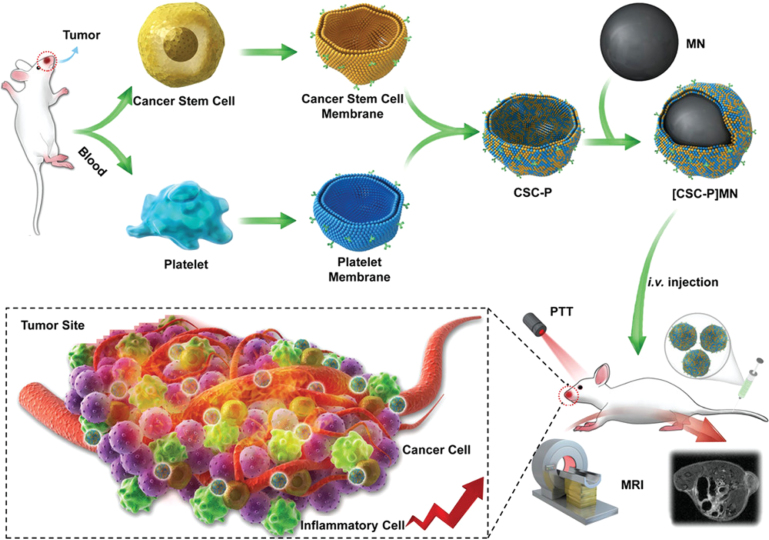
Fabrication of HCMs-NPs from CSCs and PLTs. Membrane material is derived from both CSCs and PLTs, then fused by sonication and extrusion. The resulting fused HCM is coated onto Fe_3_O_4_ magnetic NPs . These HCMs-NPs, after systemic circulation, benefit from the homotypic targeting effect at the tumor site, enhancing *in vivo* tumor magnetic resonance imaging and PTT.

Chen et al. developed DOX-loaded PLGA NPs coated by HCMs from RAW264.7 macrophage cells and 4T1 breast cancer cells to treat lung metastases from breast cancer. HCM-PLGA NPs displayed the multi-targeting abilities of hybrid membranes and were able to treat lung metastases originating from breast cancer [150]. Bu et al. developed a novel phototherapy cancer treatment platform by combining PLTs and CSCs-CMs to coat iron oxide magnetic NPs (Fig. 11). These HCMs-NPs significantly outperformed NPs coated with single-CMs in delivering photothermal treatment for head and neck squamous cell carcinoma [145].

Combinatorial therapies are commonly employed with HCMs-NPs. Wang et al. developed an HCMs-NP from RBCs and melanoma cell (B16-F10) CMs, coating DOX-loaded hollow CuS NPs. The prepared HCMs-NPs exhibited a high tumor-targeting capability, significantly enhancing photothermal and chemotherapy effectiveness [151]. Similarly, Huo et al. engineered HCMs from RBCs and MCF-7 cancer CMs to coat DOX-loaded hollow AuNPs, combining hyperthermia and controlled drug release [80]. Wang et al. also developed an innovative cancer therapy by coating hollow polydopamine NPs with a fusion of bacterial and B16-F10 melanoma CMs. Intravenously injected, these HCMs-NPs accumulate precisely at tumor sites, demonstrating sustained tumor suppression and complete eradication of melanoma without adverse effects, illustrating a promising synergistic approach in cancer therapy [40]. Interestingly, Huang et al. developed triple HCMs with CMs from glioblastoma, macrophage, and microglia cells. Supramolecular micelles encapsulating the chemotherapeutic agent MTIC, known for its efficacy in glioblastoma treatment, were coated with this triple-hybrid membrane. This multifaceted coating enhances the micelles’ ability to cross the blood–brain barrier and target glioblastoma tissues more efficiently than single-membrane-coated systems [144].

**Table 6:** NPs coated with HCMs for tumor management. CSCs: Cancer Stem Cells, DOX: Doxorrubicin, ICG: indocyanine green, MTIC: methyl-triazeno-imidazole-carboxamide. When the targeted organ is not specified, it corresponds to the tumor itself.

Cell type	NP	Cargo	Application	Disease / Targeted organ	Reference
RBCs–Cancer (Melanoma)	CuS NPs	DOX	Chemo- and Phototherapy	Melanoma	[151]

RBCs–Cancer (Ovarian cancer)	Iron Oxide NPs	ICG	Photo- and Immunotherapy	Ovarian cancer	[152]

Bacterial-Cancer (Melanoma)	Polymeric NPs		Photo- and immunotherapy	Melanoma	[40]

Macrophage-Cancer (Breast cancer)	PLGA NPs	DOX	Chemotherapy	Lung metastasis (breast cancer)	[150]

RBCs-Cancer (Breast cancer)	Gold NPs	DOX	Chemo- and Phototherapy	Breast cancer	[80]

CSCs-Platelet	Iron Oxide NPs		Phototherapy	Human squamous carcinoma	[145]

Microglia-Macrophage-Cancer (Glioblastoma)	Micelles	MTIC	Chemotherapy	Glioblastoma multiforme	[144]

[145].

## 4 CMs-NPs under clinical trials

Despite the potential of CMs-NPs, their clinical translation in oncology remains limited, mainly due to the scalability issues previously mentioned. A literature search was conducted in the Cochrane Library and ClinicalTrials, focusing on CMs-NPs as a cancer treatment. This research yielded only two results: the clinical trials NCT02657460 and NCT01854866, both of which are closed [153], [154]. These studies aimed to investigate the anticancer effects of tumor cell-derived microparticles packaging chemotherapeutic agents in the treatment of malignant ascites and malignant pleural effusion. It should be noted that this work does not specifically include CMs-NPs, as it focuses on cell membrane-derived vesicles generated by isolating, fragmenting, and purifying membranes from autologous tumor cells [155].

In 2023, Cello Therapeutics, Inc. received FDA approval for its CMs-NPs immunotherapy, CE120, to enter clinical trials. CE120 employs a platelet cell membrane coating on polymeric nanoparticles carrying a potent immunotherapy drug, the TLR agonist resiquimod (R848). This nanoparticle formulation achieves targeted delivery and prolonged residence within tumor tissues after local administration. Studies demonstrated strong local immune activation, leading to complete tumor regression in a colorectal cancer model. In an aggressive breast cancer model, intratumoral administration significantly delayed tumor growth and inhibited lung metastasis [107]. According to Cello Therapeutics, CE120 is currently in Phase I, although it is not yet listed in public clinical trial databases. Additionally, the company is developing other CMs-NPs-based products that remain in preclinical testing [156]. Therefore, there is a clear need for the clinical translation of this technology to overcome existing limitations. Key challenges include maintaining sterility throughout the production process, understanding the pharmacokinetics and biodistribution of CMs-NPs *in vivo*, and addressing issues of reproducibility and scalability. Although CMs-NPs present a promising platform, they remain a novel technology that requires further clinical validation.

CMs-NPs have also demonstrated promise beyond oncology. The clinical trial NCT 06050993, opened in September 2023, is investigating the effects of platelet-mimicking nanoparticles in patients with cirrhosis to assess their safety and efficacy in managing thrombocytopenia and related complications [157]. In the infectious disease field, Cellics Therapeutics has developed CTI-005, a human RBC nanosponge designed for patients hospitalized with Staphylococcus aureus pneumonia. By leveraging natural receptors on RBC membranes, CTI-005 binds bacterial toxins, enabling the immune system to eliminate pathogens more effectively [15], [16], [158], [159]. Similarly, another innovative approach developed by this company is CTI-111, a macrophage nanosponge designed to treat sepsis and other conditions. These nanosponges utilize natural receptors on macrophage membranes to absorb bacterial toxins and inflammatory cytokines, potentially preventing the progression of sepsis [17], [158], [160].

These ongoing advancements highlight the versatility of CMs-NPs, extending beyond oncology, demonstrating their potential to revolutionize various fields of clinical application. However, important challenges must still be addressed to ensure their successful translation into clinical practice.

## 5 Conclusions and future remarks

In this review, we explore CMs-NPs designed for tumor treatment, highlighting their potential in cancer therapeutics. By employing a top-down strategy that utilizes natural cell components, this approach offers an alternative to traditional bottom-up methods, enhancing the biointerfacing capabilities of NPs. Initially developed to extend the circulation time of NPs with RBCs coatings, this technique has expanded to include various cell types such as PTLs, white blood cells, cancer cells, and bacteria, alongside a diverse array of nanoplatforms composed of materials like polymers, lipids, and metals. The inherent biological interactions of CMs placed on the NP’s surface result in reduced nonspecific uptake and improved tumor targeting. Furthermore, the versatility of these cell types, each with unique functional traits, combined with the adaptability of NP designs, enables the creation of highly specialized platforms for targeted drug delivery, phototherapy, immunotherapy, and diagnostic imaging.

Despite significant progress, several challenges must be addressed to fully harness the potential of CMs-NPs. Achieving an optimal and complete coating remains a significant issue, as existing characterization methods as TEM, DLS, and SDS-PAGE provide limited quantitative insight into the variability and completeness of CM coatings. Innovations such as an assay to quantify coating completeness and the introduction of synthetic phospholipids to improve the coating process, represent promising advancements toward achieving more reliable CMs-NPs.

Looking ahead, translating these biomimetic platforms into clinical settings involves overcoming substantial challenges related to scalability and manufacturability. Challenges such as sourcing sufficient quantities of CMs – whether from autologous sources or type-matched donors – must be solved. Additionally, developing high-yield methods for CMs-derivation and scalable coating techniques is essential for clinical applications. Notably, the use of helper lipids developed by Lehto’s team could prove beneficial, as it significantly reduces the amount of CMs needed to produce CMs-NPs with enhanced biointerfacing properties. This intermediate platform can also improve the development of HCMs coatings. Ensuring NPs purity and developing effective filtration and storage protocols are also critical steps that need optimization. As the field of CMs-NPs continues to evolve, efforts to refine these technologies and transition them from the laboratory to clinical applications are expected, ultimately allowing CMs-NPs to be used in the clinic and revolutionize treatment methodologies.

## CRediT authorship contribution statement

**Pablo Graván:** Writing – original draft, Writing – review & editing, Visualization, Project administration, Investigation, Formal analysis, Conceptualization. **Juan Antonio Marchal:** Writing – review & editing, Supervision, Resources, Project administration, Funding acquisition, Conceptualization. **Francisco Galisteo-González:** Writing – review & editing, Supervision, Resources, Project administration, Funding acquisition, Conceptualization.

## Declaration of Generative AI and AI-assisted technologies in the writing process

During the preparation of this work the authors used OpenAI in order to improve readability and language of the work. After using this tool/service, the authors reviewed and edited the content as needed and take full responsibility for the content of the publication.

## Declaration of competing interest

The authors declare that they have no known competing financial interests or personal relationships that could have appeared to influence the work reported in this paper.

## Data Availability

No data was used for the research described in the article.
